# Reducing Postoperative Recurrence of Early‐Stage Hepatocellular Carcinoma by a Wound‐Targeted Nanodrug

**DOI:** 10.1002/advs.202200477

**Published:** 2022-05-07

**Authors:** Bozhao li, Xiuping Zhang, Zhouliang Wu, Tianjiao Chu, Zhenlin Yang, Shuai Xu, Suying Wu, Yunkai Qie, Zefang Lu, Feilong Qi, Minggen Hu, Guodong Zhao, Jingyan Wei, Yuliang Zhao, Guangjun Nie, Huan Meng, Rong Liu, Suping Li

**Affiliations:** ^1^ CAS Key Laboratory for Biomedical Effects of Nanomaterials and Nanosafety CAS Center for Excellence in Nanoscience National Center for Nanoscience and Technology Beijing 100190 China; ^2^ College of Pharmaceutical Science Jilin University Changchun 130021 China; ^3^ Faculty of Hepato‐Biliary‐Pancreatic Surgery Chinese People's Liberation Army (PLA) General Hospital Institute of Hepatobiliary Surgery of Chinese PLA Key Laboratory of Digital Hepatobiliary Surgery PLA Beijing 100853 China; ^4^ National Cancer Center/National Clinical Research Center for Cancer/Cancer Hospital Chinese Academy of Medical Sciences and Peking Union Medical College Beijing 100021 China; ^5^ University of Chinese Academy of Sciences Beijing 100049 China; ^6^ Center of Materials Science and Optoelectronics Engineering University of Chinese Academy of Sciences Beijing 100049 China; ^7^ GBA Research Innovation Institute for Nanotechnology Guangzhou 510530 China

**Keywords:** combination therapy, hepatocellular carcinoma recurrence, mesoporous silica nanoparticle, platelet membrane

## Abstract

New strategies to decrease risk of relapse after surgery are needed for improving 5‐year survival rate of hepatocellular carcinoma (HCC). To address this need, a wound‐targeted nanodrug is developed, that contains an immune checkpoint inhibitor (anti‐PD‐L1)and an angiogenesis inhibitor (sorafenib)). These nanoparticles consist of highly biocompatible mesoporous silica (MSNP) that is surface‐coated with platelet membrane (PM) to achieve surgical site targeting in a self‐amplified accumulation manner. Sorafenib is introduced into the MSNP pores while covalently attaching anti‐PD‐L1 antibody on the PM surface. The resulting nano‐formulation, abbreviated as a‐PM‐S‐MSNP, can effectively target the surgical margin when intraperitoneally (IP) administered into an immune competent murine orthotopic HCC model. Multiple administrations of a‐PM‐S‐MSNP generate potent anti‐HCC effect and significantly prolong overall mice survival. Immunophenotyping and immunochemistry staining reveal the signatures of favorable anti‐HCC immunity and anti‐angiogenesis effect at tumor sites. More importantly, microscopic inspection of a‐PM‐S‐MSNP treated mice shows that 2 out 6 are histologically tumor‐free, which is in sharp contrast to the control mice where tumor foci can be easily identified. The data suggest that a‐PM‐S‐MSNP can efficiently inhibit post‐surgical HCC relapse without obvious side effects and holds considerable promise for clinical translation as a novel nanodrug.

## Graphical abstract

a‐PM‐S‐MSNP nanodrug can effectively target the surgical margin, thus generating anti‐HCC immunity and anti‐angiogenesis effect at tumor sites. Excitingly, the a‐PM‐S‐MSNP can efficiently inhibit post‐surgical HCC relapse without obvious side effects and holds considerable promise for clinical translation as a novel nanodrug.

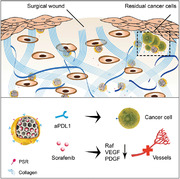

## 1 Introduction

**Figure 1:**
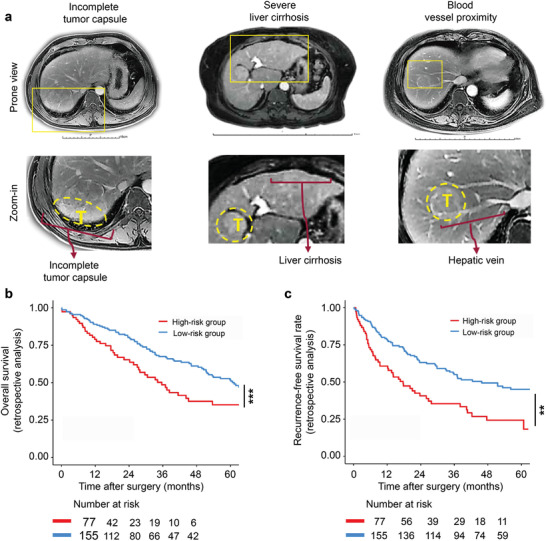
Clinical characteristics of HCC patients with high‐risk postoperative recurrence. (a) Three representative MRI images show the unfavorable risk factors that may associate with incomplete HCC removal. It includes incomplete tumor capsule of HCC (left panel), severe liver cirrhosis (middle panel) and HCC adjacent to the great vessels (right panel). (b, c) A total of historical 232 patients were categorized into low vs high risk groups using the criteria discussed in (a). Kaplan–Meier analyses revealed statistically improved overall survival (OS) (b) and recurrence‐free survival (RFS) (c) in the low risk group compared to the high risk group. Significance analyses were performed using log‐rank test (b, c). The *p* values were *p* < 0.001 and *p* < 0.01 for OS and RFS, respectively. *p < 0.05, **p < 0.01, ***p < 0.001. The striking clinical difference highlights the necessity to develop novel therapy to address HCC recurrence with a view to improve the survival outcome.

Hepatocellular carcinoma (HCC) is a leading cause of cancer death worldwide, especially in the developing countries.^[^ ^1^ ^]^ While the “curative surgery” is an ideal option for early‐stage HCC, approximately 70% postoperative patients suffer from tumor relapse within five years.^[^ ^2^ ^]^ It was generally accepted that recurrence is primarily due to the residual HCC tumor cells resided in the surgical margins, or through intraoperative hemorrhage that creates the opportunity for cancer cell escape into the circulation.^[^ ^3^ ^]^ Furthermore, when the cancerous tissue is localized to a specific area of liver, the surgery procedure per se is technically challenging. To conceptualize the complexity, we provided the magnetic resonance imaging (MRI) images from three representative patients wherein the microscopic residual tumors may be left behind after surgery. It includes: 1) highly invasive HCC with incomplete capsule, 2) severe liver cirrhosis, and 3) HCC tumor burden that is adjacent to large blood vessels (**Figure** **1**a). While incomplete cancer removal is a reasonable compromise to lower the risk of hemorrhage or potential liver failure, an obvious drawback is the high likelihood of cancer recurrence. To address the problems in these “risky” cases so as to improve HCC therapeutic efficacy, we developed a nano‐enabled approach utilizing a wound‐targeted nanocarrier, which contains an immune checkpoint inhibitor and a multi‐kinase and angiogenesis inhibitor sorafenib, for targeted therapy for these unique populations.

According to FDA, sorafenib, as the most classic approved drug for systemic treatment of HCC, only benefits limited patients in terms of survival outcome.^[^ ^4^ ^]^ Encapsulating this drug for targeted delivery is expected to provide a sustained and compartmentalized drug concentration at tumor sites, and thus improves the therapeutic effect of sorafenib and in vivo safety. In addition, we were inspired by a recent preclinical discovery, demonstrating that sorafenib improved the efficacy of anti‐PD‐1 antibody in a murine BNL‐MEA HCC model, in which immune checkpoint inhibitor treatment yielded minimal benefit alone.^[^ ^5^ ^]^ Anti‐angiogenic drugs can significantly improve the tumor microenvironment and facilitate the effects of PD‐1/PD‐L1 monoclonal antibodies. Further, PD‐1/PD‐L1 monoclonal antibodies also contribute to anti‐tumor angiogenesis, complementing each other to exert a synergistic effect.^[^ ^6^ ^]^ These facts prompt us to combine these two drugs in one nanoplatform to maximize therapeutic efficacy for HCC. Multifunctional mesoporous silica nanoparticles (MSNP) have recently received substantial attention as drug carriers due to their advantages of high drug loading capability, good biocompatibility, high internal surface area and pore volume, colloidal stability and the feasibility of specific functionalization on the external particle surface, all of which collectively afford the material great potential for use in clinical applications.^[^ ^7^ ^]^ We therefore constructed a mesoporous silica nanoparticle (MSNP) capable of co‐delivering PD‐L1 antibody and sorafenib that is suitable for intraperitoneal (IP) administration. To achieve active targeting to the surgical site, we coated platelet membrane (PM) on the MSNP surface, which is enriched in membrane surface receptors (e.g., GPIb, GPIa‐IIa, CD41, and CD61) being able to bind to collagen IV exposed upon the surgical wound.^[^ ^8^ ^]^ PM not only bestows nanoparticles with an excellent wound‐site targeting, but also protects nanoparticles from immune system attack and rapid clearance.^[^ ^8^, ^9^ ^]^ More importantly, PM itself can also bind to each other, making the nanoparticles to accumulate at diseased sites in a self‐amplified manner. Our data demonstrate that the resulting nanodrug, a‐PM‐S‐MSNP, showed significant accumulation at tumor resection/bleeding sites in a stringent and immunocompetent murine model that mimics HCC recurrence. Use of a‐PM‐S‐MSNP exhibited strong efficacy in inhibiting HCC recurrence, outperforming various control treatments such as free drug combination or single drug loaded nanoparticles. Such a strategy paves the way for nanodrug development for efficient inhibition in HCC relapse and prolonging patient survival.

## 2 Results and Discussion

By revisiting the HCC cases that underwent surgical procedure from 2012 to 2015 with 5‐year follow‐up in the Chinese People's Liberation Army (PLA) General Hospital, a retrospective analysis was conducted. We defined a given patient with any unfavorable feature as “risky” case as outlined in Figure 1a, which likely correlated to residual HCC and disease relapse. By viewing the historical MRI images involving 232 cases, 77 patients were characterized as “high risk” (red line in Figure 1b) while the rest 155 cases were identified as “low risk” patients (blue line in Figure 1b). Detailed patient information appears online (Table S1, Supporting Information). As expected, we were able to show statistical significance between “high” versus “low” risk patients with respect to the overall survival (OS, *p* < 0.001) and recurrence‐free survival rate (RFS, *p* < 0.01) (Figure 1b,c).

**Figure 2:**
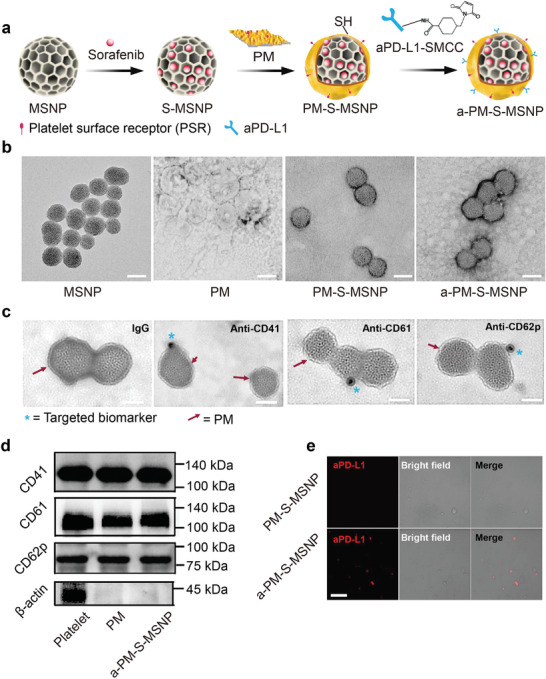
Characterization of various nanoparticles. a) Schematic illustration of the synthesis process of a‐PM‐S‐MSNP nanodrug. b) TEM images of MSNP, PM, PM‐MSNP, and a‐PM‐S‐MSNP. Negative staining created enough contrast to show the success of membrane coat. Scale bars, 100 nm. c) Immunogold staining of essential PM proteins on a‐PM‐S‐MSNP surfaces, including CD41, CD61, and CD62p. The image also provided high‐resolution details to confirm the PM coating and porosity in the MSNP samples. d) Western blot analysis to confirm the protein expression in purified platelets, platelet membranes, and a‐PM‐S‐MSNP. e) Confocal immunofluorescence experiment confirming the success of aPD‐L1 attachment in a‐PM‐S‐MSNP surfaces. Detailed experimental procedure was provided online. Scale bars, 20 µm.

This striking patient survival difference prompted us to develop an intraperitoneally (IP) injectable targeted nanodrug to provide efficacious disease management for the postsurgical HCC. We developed a platelet membrane (PM) coated nanocarrier, using mesoporous silica nanoparticle (MSNP) as nanocore structure.^[^ ^10^ ^]^ Owing to the large interior surface of MSNP and possibility of antibody attachment on the PM coat, we introduced sorafenib (a multi‐kinase inhibitor that is prescribed for advanced HCC) into the MSNP pores while covalently attaching a checkpoint inhibitor antibody, anti‐PD‐L1, on the PM surface. Briefly, MSNP particles were prepared using a standard sol‐gel chemistry procedure (**Figure** **2**a).^[^ ^10a^ ^]^ Sorafenib soaked MSNP sample (S‐MSNP) was subjected to PM coating under sonication. Note that the PM was prepared through a freeze–thaw process using a published protocol with minor modification.^[^ ^11^ ^]^ In order to achieve antibody conjugation, we employed a green chemistry procedure to first generate thiolated PM‐S‐MSNP using Traut's reagent.^[^ ^12^ ^]^ The resulting samples were used for —SH quantification, demonstrating an SH concentration of ≈150 µmol g^−1^ before antibody conjugation (Figure S1, Supporting Information). A bifunctional maleimide linker (sulfo‐SMCC) was then used to attach anti‐PD‐L1 antibody to PM‐S‐MSNP, which provided maleimide groups to react with ‐SH on PM‐S‐MSNP surfaces through the Michael addition reaction and NHS groups to conjugate with —NH_2_ from antibody.^[^ ^13^ ^]^ The successful conjugation was evidenced by the fact that the —SH density was decreased to ≈50 µmol g^−1^ after antibody modification (Figure S1, Supporting Information). The results of drug loading experiments showed that MSNP has a high drug loading ability, the ratio of sorafenib to anti‐PDL1 in the a‐PM‐S‐MSNP is about 3:1, is consistent with the dose ratio of clinical (Table S2, Supporting Information). Negative staining using acetate double oxygenic uranium dye showed the success of PM coating; the high resolution transmission electron microscopy (TEM) also revealed the porous MSNP structure (Figure 2b). Dynamic light scattering data demonstrated a hydrodynamic size of ≈100 nm in PBS for the final product, referred as “a‐PM‐S‐MSNP,” with a moderate zeta potential value of approximately −20 mV in the aqueous solution (Figure S2a,b, Supporting Information). The size and zeta potential values (in brackets) for bare MSNP and PM‐MSNP were 90.6 nm (21.27 mV) and 99.8 nm (−18.4 mV), respectively. While we deliberately selected a “green chemistry” procedure to avoid the use of high temperature or harsh chemical reagents, it was critical to look at protein biomarkers that are present in the PM.^[^ ^13^ ^]^ Colloidal gold imunological and western blot assays showed the expression of PM membrane proteins after coating to MSNP surfaces (Figure 2c,d), that is, CD41, CD61, and CD62p which are crucial for platelets binding or adhesion to the wound/bleeding sites, which in this case was HCC surgical margin.^[^ ^14^ ^]^ 2D gel electrophoresis followed by Coomassie brilliant blue staining further revealed the presence of the essential membrane proteins in a‐PM‐S‐MSNP compared to various control lanes (Figure S3, Supporting Information).^[^ ^15^ ^]^ Moreover, a‐PM‐S‐MSNP could attach to gold nanoparticle beads that were conjugated with indicated wound‐related biomarker proteins (stars in Figure 2c), however, no such finding was observed using IgG‐labelled gold beads. Since we used a rat‐derived anti‐mouse PD‐L1 antibody for conjugation with PM, we incubated our particles with the Alexa Fluor 633 labeled goat anti‐rat IgG antibody to confirm the presence of antibody attachment, according to a published protocol.^[^ ^13^ ^]^ The fluorescent imaging experiment and flow cytometry analysis confirmed the success of anti‐PD‐L1 modification (Figure 2e; Figure S4, Supporting Information). We also tested the drug release profile of sorafenib in a‐PM‐S‐MSNP in PBS supplemented with 10% FBS at 37 °C, demonstrating ≈75% drug release at 24 h (Figure S5, Supporting Information). The efficient sorafenib loading allowed us to interpret the potent killing effect in a‐PM‐S‐MSNP treated tumor vascular endothelial cells compared to empty nanocarrier, a‐PM‐MSNP (Figure S6, Supporting Information).

**Figure 3:**
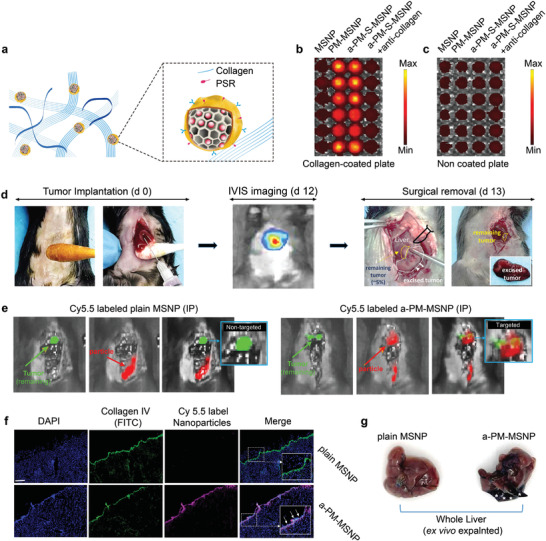
In vitro and in vivo experiments to reveal the surgical margin targeting effect of a‐PM‐S‐MSNP. a) Schematic of binding between a‐PM‐S‐MSNP and collagen. Platelet membrane coated on particle surfaces can bind to the exposed collagen of surgical margin largely through the platelet surface receptor (PSR) such as GPIb, GPIa‐IIa, CD41, and CD61. Representative imaging data of in vitro binding in b) collagen coated versus c) non‐coated plates for a‐PM‐S‐MSNP and various controls. Our data revealed a strong binding affinity between a‐PM‐S‐MSNP and collagen IV coated plates (bright red). Each treatment was repeated six times (*n* = 6). d) Establishment of surgical removal model in orthotopic HCC bearing mice. 50 µL of 5 × 10^5^ Hep1‐6 cells expressing luciferase were injected into the left lobe of the liver of anesthetized mice. Approximately 12‐day post‐inoculation, surgery removal to ≈95% tumor burden orthotopically was performed. These mice were used to study nanodrug biodistribution and efficacy in the following experiments. e) Targeting effect of Cy5.5 labeled plain MSNP or a‐PM‐MSNP after IP injection at 30 mg kg^−1^ in the mice from (d). Eight hours post‐injection, the signals of nanoparticles (red) and tumor tissues (green) were obtained. a‐PM‐MSNP gave obvious tumor lesion targeting compared with the control. f) Immunofluorescence staining of surgical margin region of the mice from (e). a‐PM‐MSNP (red) and collagen IV (green) showed obvious co‐localization, suggesting an excellent targeting ability of nanodrug for the surgical sites. g) Targeting effect of Evans blue loaded a‐PM‐MSNP for the wound. Dark bluish color pattern indicates dye accumulation in the a‐PM‐MSNP but not plain MSNP group.

In our platform, a key design feature was to use the intrinsic binding affinity between PM receptors and exposed collagen IV at the wound site as a targeting principle to locate the remaining post‐surgical HCC malignancy (**Figure** **3**a). To prove this hypothesis, a comparative experiment in vitro was first performed in 96‐well plate w/wo collagen IV coating. Fluorescently labeled particle samples were added into these wells for 2 min, followed by intensive washing steps using PBS. While the PM‐containing samples, that is, PM‐MSNP and a‐PM‐S‐MSNP, exhibited significant attachment in the collagen‐coated wells, the attachment became minimal in the plain MSNP group (Figure 3b; Figure S7a, Supporting Information). Moreover, the addition of anti‐collagen IV antibody significantly blocked this binding effect. When repeated the experiment using non‐coated 96‐well plate, the PM‐containing particle binding was neglectable across the board (Figure 3c; Figure S7b, Supporting Information).

**Figure 4:**
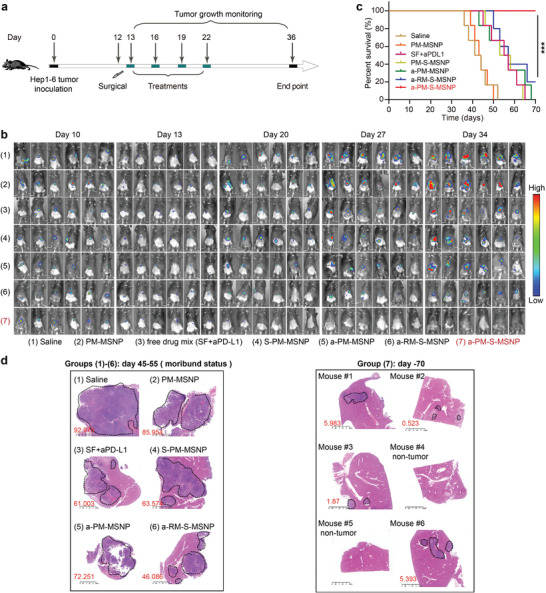
Antitumor efficacy in vivo. a) Schematic illustrating the schedule in therapeutic experiments with the orthotopic HCC surgical removal mouse model. It involved four stages: orthotopic tumor implantation (d1), tumor establishment check (d10), surgical removal (d12), and animal treatment. A total of four IP injections of a‐PM‐S‐MSNP (Sorafenib: 30 mg kg^−1^; antibody: ≈10 mg kg^−1^) and various controls were performed during day 13–22 at an interval of three days (*n* = 6). IVIS imaging begun at day 10 and ceased at day 34. b) In vivo bioluminescence imaging of orthotopic HCC after removal of the primary tumors (*n* = 6). a‐PM‐S‐MSNP group exerted the strongest anti‐HCC effect compared to other controls. c) Cumulative survival data recorded up to 70 days (*n* = 6). Mantel‐Cox Log‐rank test was conducted to show the statistical significance in the Kaplan–Meier plot, *p* < 0.001. *p < 0.05, **p < 0.01, ***p < 0.001. d) H&E staining of liver sections from mice administered with the indicated treatments. 2 out 6 were histologically tumor‐free in a‐PM‐S‐MSNP treated mice. Tumor foci were indicated in the circles. Besides, the tumor invaded liver area was calculated in the H&E staining images. Scale bars, 2.5 mm.

We continued to test the wound targeting ability of our samples in a postsurgical HCC mouse model, which was established through orthotopic inoculation of Hep1‐6 cells into C57BL/6 mice (Figure 3d). For ease of cancer visualization, we introduced luciferase‐expressing gene into Hep1‐6 cells. Approximately 12‐day post‐inoculation, it was possible to detect orthotopic HCC burden under an IVIS imager. One day later (day ≈13), a surviving animal surgery procedure was performed with an intention to remove the majority of HCC tumor burden. To mimic the “high” risk factor patients in the clinic, we purposely left ≈5% v/v HCC tumor mass in the mice while the excised HCC tumor was about ≈95% v/v. In order to ensure the minimal damage to the mice, we limited the surgical procedure to ≈10 min per animal. After abdominal closure, the tumor‐bearing mice were IP injected with either a Cy5.5 fluorescently labeled or an Evan Blue laden a‐PM‐MSNP at a particle dose of 200 mg kg^−1^. For the first setting of experiment, it was possible to capture persudo red (Cy5.5 particle) and green (tumor luciferase) signals in situ using an IVIS imaging system. Six hours post IP injection, we demonstrated co‐localization of particle and tumor signals in the same animal, an indicative of tumor targeting effect in vivo (Figure 3e, right panel). We indeed saw weak particle signal in the abdominal cavity. This contrasted to the particle distribution experiment in the non‐targeted particle group in which no obvious co‐localization was observed (Figure 3e, left panel). We then sacrificed the mice and harvested the whole liver for histology analysis. The results demonstrated that a‐PM‐MSNP had an excellent collagen IV targeting effect at the surgical margin, evidenced by the immunofluorescence co‐localization analysis (Figure 3f). In addition, we repeated the essential component of the biodistribution study using an Evans Blue laden a‐PM‐MSNP. This allowed us to obtain visual evidence to show a contrast staining pattern. Dark bluish color was observed in the a‐PM‐MSNP but not the non‐targeted MSNP group (Figure 3g; Figure S8, Supporting Information), confirming the effective accumulation of PM‐containing particles in the surgical region. After that, we evaluated the blood pharmacokinetics of a‐PM‐MSNP. The cy5.5‐labeled nanodrugs were administered and the blood drug concentration was detected as a function of time post‐injection. We acquired the blood samples at the different time points for fluorescence quantification analysis. The a‐PM‐MSNP pharmacokinetic curves suggested that exhibited a prolonged blood circulation time, compared to other control groups (Figure S9, Supporting Information).

a‐PM‐S‐MSNP nanoparticles were next evaluated for their antitumor activity, with the experiment outline appearing in **Figure** **4**a. The whole animal experiment was carried out for up to 70 days. A total of four IP injections of a‐PM‐S‐MSNP (Sorafenib: 30 mg kg^−1^; antibody: ≈10 mg kg^−1^) were performed during day 13–22 with an interval of 3 days (*n* = 6 per group). IVIS imaging begun at day 10 and ceased at day 36 owing to the animal loss or the formation of malignant ascites (that may interfere IVIS imaging). Figure 4b showed an integrated IVIS imaging data which provided the visual evidence on the tumor burden in different groups. It was clear that a‐PM‐S‐MSNP (group 7) exerted the strongest anti‐HCC effect compared to control treatments, including free drug mixture (group 3), single drug‐loaded nanoparticles (groups 4–5), and dual‐delivery particles coated with red blood cell membrane (group 6). Starting from day ≈36, we observed animal loss in the saline and empty particle (PM‐MSNP) groups due to the spontaneous animal death or moribund status that triggered euthanasia according to the approved animal welfare policy. At day 70, Kaplan‐Meier plot and the statistics indicated that a‐PM‐S‐MSNP outperformed other treatment groups in prolonging animal survival (Figure 4c, Mantel‐Cox Log‐rank test, *p* < 0.0001). Furthermore, we performed H&E staining to identify the microscopic tumor foci in all the 6 “animal survivors” in group 7 at day 70. The results showed that 2 out of 6 mice were tumor‐free in the livers, whereas all the samples from groups 1–6 collected at early time points, that is, day 45–55, showed discernable HCC characteristics (Figure 4d).

**Figure 5:**
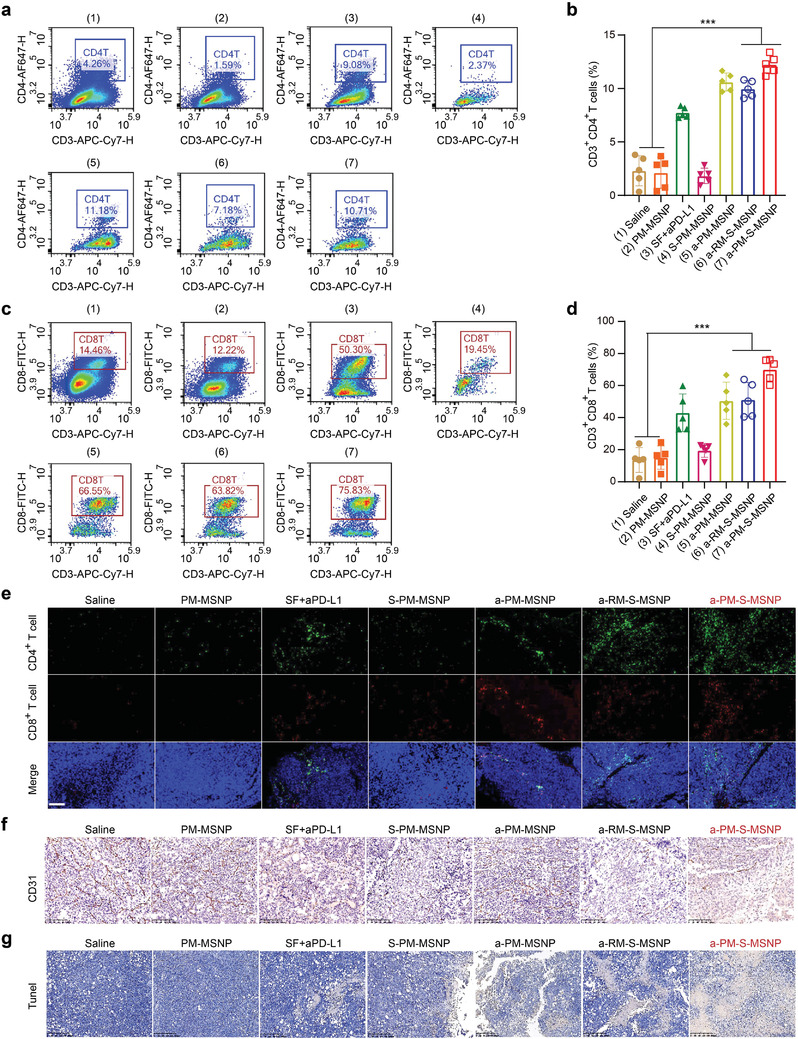
Immunophenotyping and immunofluorescent staining for a‐PM‐S‐MSNP‐ and the controls‐treated tumors. Flow cytometry analysis to reveal the abundance of a,b) CD4^+^ T cells and c,d) CD8^+^ T cells at tumor sites (*n* = 5) after administrations of a‐PM‐S‐MSNP (Sorafenib: 30 mg kg^−1^; antibody: ≈10 mg kg^−1^) and various controls. a‐PM‐S‐MSNP treatment incurred the strongest immune response across the board. Each symbol represents an individual mouse and the significance analyses were performed using one‐way ANOVA (b, d). *p < 0.05, **p < 0.01, ***p < 0.001. e) Multi‐color immunofluorescence showed CD4^+^ T cells (green) and CD8^+^ T cells (red) infiltration at tumor sites. a‐PM‐S‐MSNP treatment led to the highest T cells infiltration compared to the other groups. Scale bars, 200 µm. f) CD31 staining (brown) of the dissected tumor tissues, showing notable tumor vessel deficiency in the a‐PM‐S‐MSNP treated group in contrast to other groups. Scale bars, 100 µm. g) TUNEL staining of the dissected tumor tissues, showing the highest degree of apoptosis (brown) in the a‐PM‐S‐MSNP treated group. Scale bars, 200 µm.

To explore the immunological activation of HCC microenvironment as an extension of the efficacy data, we performed experiment with a similar schedule as seen in Figure 4a. In this case, we purposely left ≈50% v/v HCC tumor mass in the mice to ensure adequate tumor samples for flow cytometry and histological assessment (*n* = 5 per group). Notably, sorafenib or anti‐PD‐L1 containing groups, such as groups 3, 5, 6, and 7, had an ability to increase the numbers of CD4^+^ (**Figure** **5**a,b) and CD8^+^ T cells (Figure 5c,d) at tumor sites. Clearly, the a‐PM‐S‐MSNP treatment had a strongest abundance in these T cells. The favorable tumor immunological microenvironment was also reflected by immunofluorescent staining in which a‐PM‐S‐MSNP was also found to lead to the highest abundance of CD4^+^ and CD8^+^ cells in tumors (Figure 5e). Furthermore, a‐PM‐S‐MSNP administration was also accompanied by significant release of IFN‐*γ* and IL‐12, in parallel with increased levels of IL‐2, TNF‐*α*, and IL‐6, which collectively contribute to the anti‐HCC immunity (Figure S10, Supporting Information). While detailed mechanism still requires in‐depth cancer biology understanding, we surmise that both sorafenib and checkpoint inhibitor contribute to anti‐HCC immunity in our study. Indeed, there are several recent clinical data demonstrating an elevated IFN‐*γ* ^+^ CD8^+^ T cell response in HCC patients on sorafenib therapy.^[^ ^16^ ^]^

To evaluate the effect of a‐PM‐S‐MSNP treatment on tumor angiogenesis, immunohistochemical (IHC) staining was preformed to detect the density of CD31‐positive vessels at the HCC sites of animals. As expected, in contrast to various controls, a‐PM‐S‐MSNP led to a substantially obvious reduction in vessel numbers; however, interestingly, we also found comparably reduced number of blood vessels in the free drug mixture treatment group (Figure 5f; Figure S11, Supporting Information). Moreover, we conducted TdT‐mediated dUTP Nick‐End Labeling (TUNEL) staining to observe apoptosis at tumor sites. The results indicate that a‐PM‐S‐MSNP treatment resulted in the strongest apoptotic effect (Figure 5g), similar to the trend in the efficacy experiment (Figure 4).

We have previously demonstrated the utility of MSNP drug carriers for systemic delivery of chemotherapeutic agents, nuclear acids, kinase inhibitors and their combinations, with improved efficacy and reduced toxicity.^[^ ^17^ ^]^ Our data are particularly promising in pancreatic and colon cancer bearing mice receiving intravenously (IV) injected MSNP nanocarriers coated by synthetic lipid bilayer, also known as silicasome. Some of the silicasome formulations have been manufactured in large quantity, that is, 120 g per batch, enabling robust in vivo efficacy study to compare with the FDA approved formulations in multiple cancer indications.^[^ ^17c^ ^]^ Based on the new advancement in the current study that involves IP injection, we were still interested in testing a PM‐membrane coated MSNP, injected IV, for systemic treatment of solid tumors. We further established a surgical removal HCC model using subcutaneous (subQ) Hep1‐6 tumors, followed by different treatments IV. In a pilot study, assessment of the biodistribution of a‐PM‐S‐MSNP demonstrated that PM coating could provide an ≈threefold increase in a residual subQ Hep1‐6 tumor sites compared to the control particle coated with red blood cell membrane (a‐RM‐S‐MSNP) (Figure S12a,b, Supporting Information). Surprisingly, a‐PM‐S‐MSNP significantly reduced tumor growth and recurrence in the subQ study (Figure S13, Supporting Information). Besides, no apparent body weight change was observed in a‐PM‐S‐MSNP treatment group during the whole treatment process (Figure S14, Supporting Information). We also found in a separate experiment that no obvious histological and organ function indicators abnormalities were discerned in the healthy mice treated by a‐PM‐S‐MSNP or other controls (Figures S15,S16, Supporting Information), which is similar to the coating using synthetic lipids.^[^ ^17c^ ^]^ While promising in both administration routes in the context of HCC, our preference is to prioritize the use of PM coated MSNP IP with the intention to maximize the wound targeting outcome while reducing off‐target side effects at intact animal level.

Although there are translational attempts to develop cell membrane coated nanocarrier (e.g., red blood cell or macrophage membranes, https://cellics.com/full‐pipeline), there are plenty works that need to be done beyond the current proof‐of‐principle research. Future study is needed to investigate the immunological compatibility using human‐derived PM and compare the outcome between murine versus human source, perhaps in the expensive humanized animal model.^[^ ^18^ ^]^ Iterative optimization is required to improve the cell membrane coated MSNP with the view to establish nano chemistry, manufacturing and controls (nano CMC) and quality control (QC), large‐scale production, batch‐to‐batch reproducibility, and purification technique.^[^ ^19^ ^]^ While we tend to prioritize the use of IP injection, if IV would become the preferred administration route, it is crucial to ensure the long‐term colloidal stability and proper dispersion techniques (e.g., suspending agents or lyophilization), which is beyond the scope of this work.

## Conflict of Interest

The authors declare no conflict of interest.

## Author Contributions

B.L., X.Z., and Z.W. contributed equally to this work. S.L., R.L., and H.M. designed the research. B.L. and X.Z. performed the experiments. T.C. and S.X. helped to draw the schematic diagram. Z.W., Y.Q., Z.L., F.Q., T.C., Z.Y., and S.W. assisted in the establishment of postoperative animal model and flow cytometry analysis. G.N. and H.M provided suggestions on data presentation. S.L., H.M., B.L., and X.Z. wrote the manuscript. G.N., Y.Z., and R.L. revised the manuscript.

## Supporting information

Supporting information

## Data Availability

The data that support the findings of this study are available in the supplementary material of this article.
