# Supplementary material for: Reducing Postoperative Recurrence of Early‐Stage Hepatocellular Carcinoma by a Wound‐Targeted Nanodrug
Source: Adv Sci (Weinh). 2022 May 7;9(20):2200477. doi: 10.1002/advs.202200477 (PMC9284141; doi:10.1002/advs.202200477)
Supplement: Supplementary file 1 — Supporting information [file ADVS-9-2200477-s001.pdf]

ADVANCED SCIENCE
Open Access

Supporting Information

for *Adv. Sci.*, DOI 10.1002/advs.202200477

Reducing Postoperative Recurrence of Early-Stage Hepatocellular Carcinoma by a Wound-Targeted Nanodrug

Bozhao li, Xiuping Zhang, Zhouliang Wu, Tianjiao Chu, Zhenlin Yang, Shuai Xu, Suying Wu, Yunkai Qie, Zefang Lu, Feilong Qi, Minggen Hu, Guodong Zhao, Jingyan Wei, Yuliang Zhao, Guangjun Nie, Huan Meng, Rong Liu* and Suping Li**

Supporting Information for

Reducing Postoperative Recurrence of Early-Stage Hepatocellular Carcinoma by a Wound-Targeted Nanodrug

Bozhao li[1,2#], Xiuping Zhang[3#], Zhouliang Wu[1#], Tianjiao Chu[1,2], Zhenlin Yang[4], Shuai Xu[3], Suying Wu[1,5], Yunkai Qie[1], Zefang Lu[1,5], Feilong Qi[1], Minggen Hu[3], Guodong Zhao[3], Jingyan Wei[2], Yuliang Zhao[1,5,6,7], Guangjun Nie[1,5,6,7], Huan Meng[1,5*], Rong Liu[3*], Suping Li[1,5,6,7*]

[1]CAS Key Laboratory for Biomedical Effects of Nanomaterials & Nanosafety, CAS Center for Excellence in Nanoscience, National Center for Nanoscience and Technology, Beijing 100190, China

[2]College of Pharmaceutical Science, Jilin University, Changchun 130021, China

[3]Faculty of Hepato-Biliary-Pancreatic Surgery, Chinese People's Liberation Army (PLA) General Hospital; Institute of Hepatobiliary Surgery of Chinese PLA; Key Laboratory of Digital Hepatobiliary Surgery, PLA, Beijing, China

[4]National Cancer Center/National Clinical Research Center for Cancer/Cancer Hospital, Chinese Academy of Medical Sciences and Peking Union Medical College, Beijing 100021, China

[5]University of Chinese Academy of Sciences, Beijing 100049, China

[6]Center of Materials Science and Optoelectronics Engineering, University of Chinese Academy of Sciences, Beijing 100049, China

[7]GBA Research Innovation Institute for Nanotechnology, Guangzhou, 510530, China

*Correspondence to: mengh@nanoctr.cn, liurong301@126.com, lisuping@nanoctr.cn

Experimental Section

Materials: Cetyltrimethylammonium bromide (CTAB), Tetraethylorthosilicate (TEOS), bovine serum albumin (BSA) and prostaglandin E1 (PGE1) were purchased from Sigma-Aldrich (Shanghai, China). Sorafenib (BAY 43-9006) was purchased from Selleck Chemicals (Shanghai, China). The anti-PD-L1 antibody was obtained from Biolegend (124329). Dulbecco's modified Eagle medium (DMEM) and phosphate buffer saline (PBS) solution were purchased from Wisent Corporation (Wisent, Canada). Antibody for CD62p (0561R) was purchased from Bioss, Ltd. Antibodies for CD41 (ab134131), CD61 (ab179473) and anti-beta actin antibody (ab8226) were purchased from Abcam. Antibodies used for flow cytometry, CD45-PerCP/CY5.5 (103132), CD3-APC/CY7 (100222), CD3-APC (100236), CD4-AF647 (100426), CD4-BV510 (100449), CD8-FITC (100706), CD44-PE/CY7 (103030), and CD62L-APC/CY (104428) were purchased from Biolegend.

Animals and Cell Lines: C57BL/6N mice were purchased from Vital River Animal Laboratories (Beijing, China). The female animals with the age of 6~8 weeks were used throughout the experiments. The animal study was approved by the Institutional Animal Care and Use Committee of the National Center for Nanoscience and Technology (NCNST). The murine hepatocellular carcinoma cells (Hep1-6) were provided by Cell Bank, Shanghai Institute of Biochemistry and Cell Biology. Human umbilical vein endothelial cells (HUVECs) were purchased from Jennio Biotech (Guangzhou, China). Hep1-6 cells were cultured in DMEM high-glucose medium with 10,000 units/mL of penicillin, 10,000 μg/mL of streptomycin and 10 % FBS. HUVECs were cultured in F-12K medium with 10,000 units/mL of penicillin, 10,000 μg/mL of streptomycin, 10 % FBS and 5 % endothelial cell growth supplement factor (ECGS) to mimic tumor associated vascular endothelial cells (T-VECs). All these cells were incubated with 5% CO_2 at 37 °C.

Preparation of Blank and Sorafenib-loaded-MSNP: Bare MSNP nanoparticles were synthesized with the previously described method.[1] In brief, 400 mg CTAB and 1.4 mL sodium hydroxide solution (2 M) were added in 200 mL deionized water and heated to 75 °C. After that, 2 mL TEOS solution was added into the CTAB-containing

solution. Four hours later, the sample was repeatedly washed in acidic ethanol for 36 hours. For drug loading, sorafenib was loaded into MSNP nanoparticles by stirring at 2000 rpm for eight hours. The suspension was then centrifuged at 10,000 rpm for 15 min to remove the unloaded drug.

Isolation and Purification of Platelet Membranes: Platelet membrane preparation was conducted according to literature with minor modification.[2] Briefly, the whole blood from mice was collected by cardiac puncture with ACD buffer as anticoagulant (V/V= 9:1, 75 mM sodium citrate, 39 mM citric acid and 135 mM dextrose, pH = 7.4). The blood was then centrifuged at 120 g for 20 min to obtain the platelet-rich plasma (PRP). PRP was then centrifuged at 800 g for 10 min to obtain platelets. For platelet membrane isolation and purification, the platelets were resuspended in Tyrode's buffer (12 mM $NaHCO_3$, 10 mM HEPES, 134 mM NaCl, 1 mM $MgCl_2$, 0.34 mM Na_2HPO_4, 2.9 mM KCl, pH = 7.4), and the suspension was subjected to five freezing (-80 °C) - thaw (25 °C) cycles and centrifuged at 12,000 g for 30 min at 4 °C to acquire the platelet membrane. To prevent platelet membrane protein degradation and platelet activation, 1 mM phenylmethylsulfonyl fluoride (PMSF) and 2 μM prostaglandin E1 (PGE1, Aladdin, China) were added during the process.

Membrane Coating and aPD-L1 Conjugation: Platelet membrane was extracted as described above and erythrocyte membrane were extracted according to the previous studies[2]. In order to explore the optimal ratio of platelet membrane to MSN, 10 mg MSNP nanoparticles were dispersed in 1 mL PBS solution that contained different amounts of platelet membranes. To coat the platelet membrane onto the surface of MSNP-S, 10 mg MSNP-S were mixed with different amounts of platelet membrane fragment. This step was done with sonication on ice (42 kHz, 100 W) for 45 min. We then confirmed the amount of platelet membrane needed to completely coat the MSNP via TEM and DLS. When no free MSNP was observed under the electron microscope, and the surface potential of the nanomedicine was the same as that of the pure platelet membrane, it was considered that all the mesoporous silicon nanoparticles were covered by the platelet membranes. By this methodology, we found an optimal formulation (MSNP:PM = 1:1.5, mass ratio). For aPD-L1 conjugation to PM-S-MSNP, nanoparticle surfaces were thiolated with Traut's reagent for 45 min. The excess Traut's reagent was removed by washing three times using Tyrode's buffer (800 g, 5 min). Next,

aPD-L1 antibody was mixed with sulfosuccinimidyl-4-(N-maleimidomethyl)-cyclohexane-1-carboxylate (Sulfo-SMCC, Pierce) in protein storing buffer at the molar ratio of 1:5 for 2 hours at 4 °C. The excess Sulfo-SMCC was discarded by dialysis (molecular weight cut-off size, 10 kDa) to obtain aPD-L1-sulfo-SMCC. Finally, the cell membrane coated MSNP was mixed with aPD-L1-sulfo-SMCC at a protein mass ratio of 1:10. Unreacted aPD-L1 antibody was removed through centrifugation at 10,000 g for 10 min to obtain a-PM-S-MSNP. During this reaction process, thiol groups density was measured with the Total Mercapto (-SH) Measurement Kit (BestBio, BB-472422).

Characterization: The final and the intermediate products were fully characterized. For the morphology observation, transmission electron microscopy (TEM, JEOL 1200-EX) was used. Different formulation nanoparticle suspensions were dropped onto the copper TEM grid (Ted Pella, CA) and negative-stained with the sodium phosphotungstate solution, then dried overnight at room temperature. Electron micrographs were imaged at 200 kV accelerating voltage. The zeta potential and size were monitored by Dynamic Light Scattering system (DLS, Zetasizer Nano ZS90, Malvern, UK). The protein profiles of platelets, PM and a-PM-S-MSNP were measured by SDS-PAGE and western blot experiments. These experiments were used to analyze whether the major functional proteins of platelet membrane (*e.g.* CD41, CD61 and CD62p) were preserved after membrane coating. First, different sample precipitates were collected by centrifugation at 15,000 g for 10 min and dissolved in high-efficiency tissue lysate buffer (RIPA) on ice for 20 min. The supernatant was collected by centrifugation at 12,000 g for 15 min and boiled in the loading buffer for 5 min. For the SDS-PAGE experiment, 10-well 10% polyacrylamide gels were used and protein separation was performed by Novex Xcell Surelock Electrophoresis System (Bio-Rad, USA). To obtain protein expression profiles of different samples, the protein gels were placed in Coomassie brilliant blue protein staining solution for 8 hours and the gels were destained in destaining solution overnight. For the western blot experiment, after SDS-PAGE, the protein gels were transferred to the poly (vinylidene difluoride) (PVDF) membranes and blocked in 5% non-fat milk for 1 h. Primary antibodies and horseradish peroxidase-conjugated secondary antibodies were used to incubate with the PVDF membrane. All bands were visualized by the Bio-Rad ChemiDox Touch Imaging System (Bio-Rad, USA). For the immunogold staining experiment, 10 μL a-PM-S-MSNP solution was placed on the copper grids and removed after incubation for 30 min

at 25 °C. Next, copper grids were washed twice with PBS (1% BSA, 50 nM glycine). After that, 10 μL CD41, CD61 and CD62p primary antibodies were added onto the copper grids, treated for 30 min at 25°C and then blocked with BSA (1%) for 20 min. Then, the samples were stained with 10 μL IgG-gold conjugate (5 nM) for one hour. Finally, the samples were fixed with 1% glutaraldehyde for 10 min and stained with 1% uranyl acetate for 10 min. The pictures of immunogold staining were obtained by TEM.

***Ex vivo* Collagen Binding Experiment:** Firstly, 100 μL of the collagen type IV solution (0.5 mg/mL, Southern Biotech) was dropped into each well of 96-well plates and placed overnight at 4 °C. Before the collagen binding experiment, collagen pre-coated plates were blocked with 2% BSA and washed twice with PBS. After that, 100 μL of 1 mg/mL Cy5.5 labeled nanoparticles were added into collagen-coated or non-coated plates (n = 6) and incubated for 2 min. Then, the plates were washed three times with PBS. The nanoparticles that remained on plates were dissolved with 100 μL DMSO for fluorescence imaging and quantification.

Establishment of Orthotopic Hepatocellular Carcinoma Resection Model: For the establishment of orthotopic hepatocellular carcinoma murine model, 50 μL of 5 x 10^5 Hep1-6 cells were injected into the left lobe of the liver of anesthetized mice. Hep1-6 tumor growth was monitored by the Maestro *in vivo* imaging system (Cri Maestro, USA). When the tumor volume reached around 200-300 mm^3, secondary surgery was performed and the primary tumors were resected, leaving about 5% residual tumor tissue to mimic postoperative positive tumor margin.

Establishment of Subcutaneous Hepatocellular Carcinoma Resection Model: For the establishment of subcutaneous hepatocellular carcinoma murine model, 100 μL of 5 × 10^6 Hep1-6 cells were injected subcutaneously into anesthetized mice. When the tumor volume reached around 300 mm^3, surgery was performed and the primary tumors were resected, leaving about 5% residual tumor tissue to mimic postoperative positive tumor margin.

Surgical Margin Targeting Experiment of Orthotopic HCC Postoperative Model: For analysis of the postoperative margin targeting ability of a-PM-S-MSNP, we introduced a dual reporter system for fluorescence and bioluminescence. Luciferase-

expressing gene was transfected into Hep1-6 cells and the residual tumor tissue was imaged *via* the bioluminescence signals (green channel). After the resection of primary tumor, different formulations of Cy5.5 loaded nanoparticles were IP injected and the distribution of Cy5.5 labeled nanoparticles was monitored *via* the fluorescence signals (red channel). We found an obvious overlap of red channel and green channel in a-PM-S-MSNP treated group but not in other groups.

Surgical Margin Targeting and Organ Biodistribution in Subcutaneous HCC Postoperative model: Mice that received surgery were intravenously injected with different formulations of Cy5.5 loaded nanoparticles at a dose of 100 nmol/kg of Cy5.5 (n = 3). After eight hours, the mice were euthanized to collect the tumor tissues and major organs (heart, spleen, liver, lung, kidney). The fluorescent signal of Cy5.5 in different tissues was measured by Maestro *in vivo* imaging system (Cri Maestro, USA).

***In vivo* antitumor effects:** For the treatment of orthotopic HCC resection model, the mice that received surgery were IP injected with saline, PM-MSNP, free drug mixture (sorafenib plus anti-PD-L1), S-PM-MSNP, a-PM-MSNP, a-RM-S-MSNP, a-PM-S-MSNP every three days for four times (Sorafenib: 30 mg/kg; antibody: ~10 mg/kg). The volumes of orthotopic HCC tumors were measured using Maestro *in vivo* imaging system at different time points. All mice were observed until euthanasia or the survival endpoint up to 70 days. The livers of the mice that was euthanized or sacrificed at the end point were removed and fixed with 4% paraformaldehyde, sectioned, and stained with haemotoxylin and eosin (H&E).

For the treatment of subcutaneous HCC resection model, the mice that received surgery were IV injected with saline, PM-MSNP, free drug mixture (sorafenib plus anti-PD-L1), S-PM-MSNP, a-PM-MSNP, a-RM-S-MSNP, a-PM-S-MSNP every three days for four times (Sorafenib: 30 mg/kg; antibody: ~10 mg/kg). Tumor volume was calculated using the following formula: (long diameter × short diameter^2)/2.

For the analysis of immunological activation of HCC microenvironment, the orthotopic HCC tumor was removed and ~50% (v/v) was left to ensure adequate tumor samples for flow cytometry and histological assessment. The mice that received surgery were IP injected with saline, PM-MSNP, free drug mixture (sorafenib plus anti-PD-L1), PM-S-MSNP, a-PM-MSNP, a-PM-S-MSNP, a-PM-S-MSNP every three days for two times. At the end point of therapy, the mice were sacrificed where their spleens and

tumor tissues were harvested for flow cytometry or immunofluorescence analysis. For flow cytometry analysis, the harvested tissues were homogenized to produce single-cell suspension following fluorescence labeling with appropriate antibodies. For the analysis of $CD8^+$ or $CD4^+$ T cells infiltration level in tumor tissues, the single-cell suspensions of tumor tissues were stained with anti-CD45-PerCP-Cy5.5, anti-CD3-APC-Cy7, anti-CD4-AF647 and anti-CD8-FITC anti-CD86-PE. The stained cells were collected and analyzed on a flow cytometer (Agilent, Quant). Meanwhile, the tumor tissues were fixed with 4% paraformaldehyde for subsequent immunohistochemical staining, including CD4 and CD8.

IHC staining: HCC tumor tissues were harvested at the same time of mice sacrifice and were fixed in 4% paraformaldehyde for 12 hours, followed by paraffin embedding and sectioning. Sections of 6-μm thickness were stained with CD31, TUNEL or haemotoxylin and eosin (H&E). The images of IHC staining were imaged by confocal laser scanning microscope (Zeiss Z-710, Germany).

Safety Evaluation: After treatment with different formulations of nanoparticles every three days for total three treatments, the mice were sacrificed and heart, lung, liver, spleen and kidney were harvested for H&E staining to study the potential changes in organ morphology.

Statistical Analysis: Results data were analyzed by SPSS 17.0 statistical analysis software. All error bars are presented as mean ± S.D. Student's t-test was used for comparison between two groups, and one-way ANOVA was applied for comparison among multiple groups, respectively, $*p < 0.05$, $**p < 0.01$, and $***p < 0.001$. Animal survival rates were analyzed with the log-rank test using GraphPad Prism 8.0.

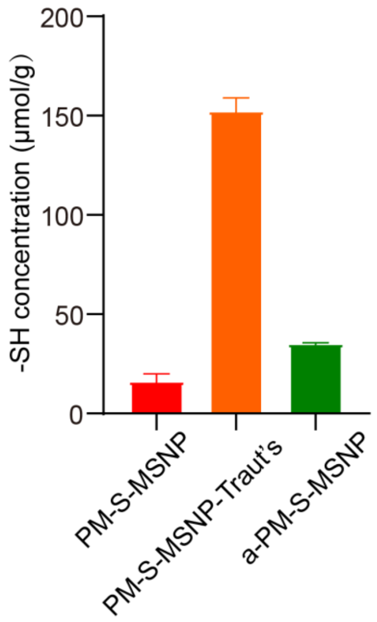

Figure S1. Thiol group quantification showing successful conjugation of anti-PD-L1 antibody. Thiol group density was measured using sulfhydryl detection kit (n = 3). The short horizontal lines indicate mean ± SD. The -SH density changed from 150 μmol/g to ~50 μmol/g after anti-PD-L1 antibody modification, indicating the successful modification of anti-PD-L1 antibody to the PM-coated drug-soaked MSNP.

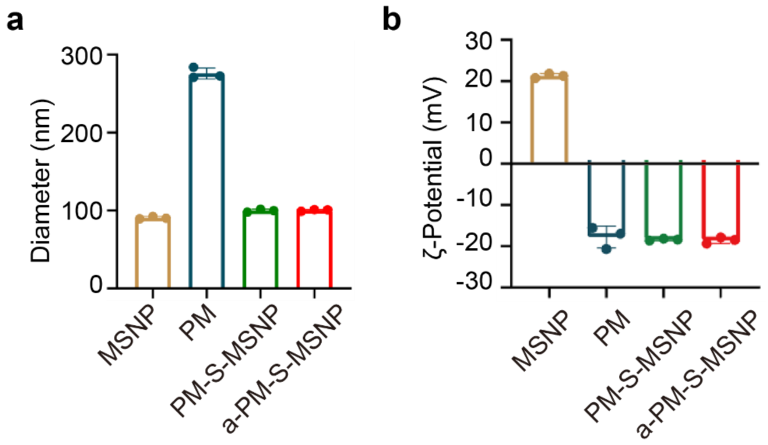

Figure S2. Hydrodynamic size (a) and zeta potentials (b) of MSNP, PM, PM-MSNP and a-PM-S-MSNP. Each symbol represents a separate experiment (n =3), the short horizontal lines indicate mean ± SD.

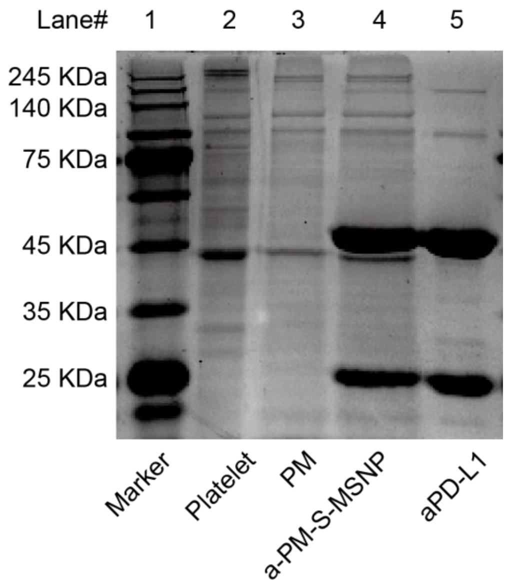

Figure S3. The presence of essential PM proteins and aPD-L1 in a-PM-S-MSNP. The protein expression profiles of fresh platelets (lane 2), platelet membranes (lane 3), a-PM-S-MSNP (lane 4) and aPD-L1 (lane 5) were analyzed using SDS-PAGE electrophoresis. a-PM-S-MSNP exhibited similar protein expression profile to platelet membrane and anti-PD-L1 (~40-60 kDa), confirming the integrity of important membrane proteins during the nanoparticle preparation and the successful antibody attachment to the platelet membrane.

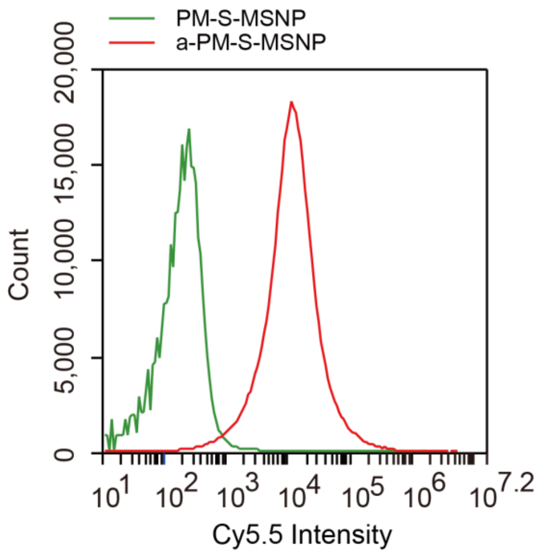

Figure S4. Flow cytometry analysis of the success attachment of aPD-L1 in a-PM-S-MSNP surfaces.

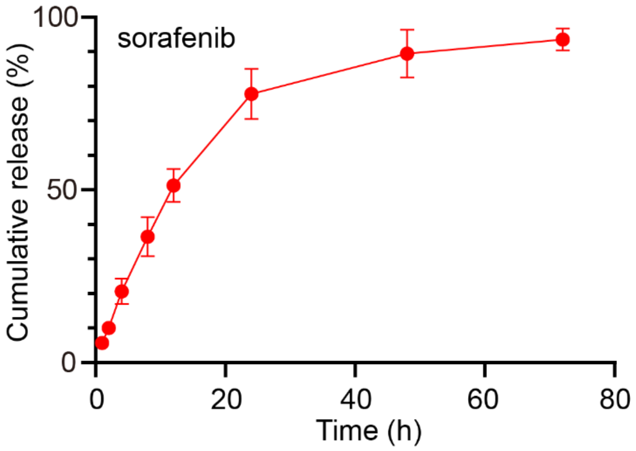

Figure S5. Cumulative release of sorafenib over time from a-PM-S-MSNP. Data are presented as mean ± SD (n = 3). The experiment was conducted in PBS at 37 °C. Sorafenib concentration was determined by high-performance liquid chromatograph (HPLC).

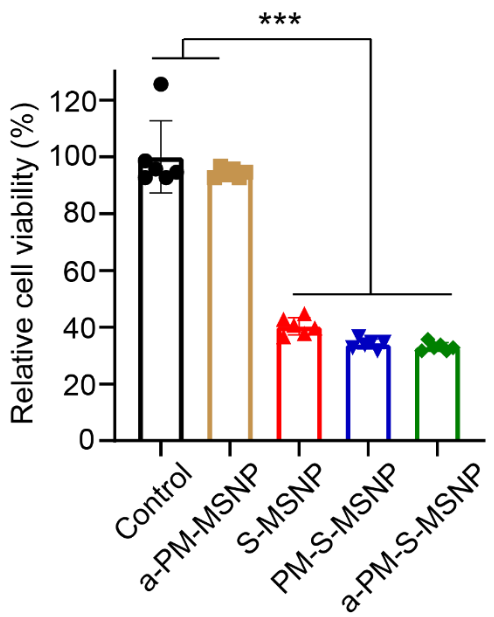

Figure S6. Cytotoxic effect of different formulations on tumor vascular endothelial cells (T-VECs). T-VECs received various nanoparticle treatments at equivalent sorafenib dose of 8 μM for 48 hours. Empty particle (a-PM-MSNP) and PBS serve as controls. All sorafenib loaded nanoparticles efficiently inhibited the proliferation of vascular endothelial cells. The short horizontal lines indicate mean ± SD (n = 6). For the significance analysis one-way ANOVA was performed. *$p < 0.05$, **$p < 0.01$, ***$p < 0.001$.

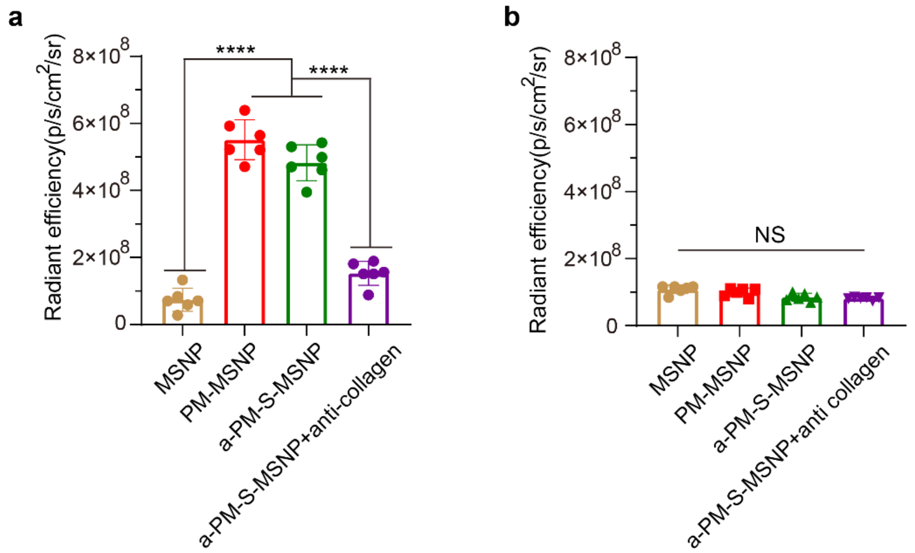

Figure S7. The quantification results of *in vitro* binding in collagen coated vs non-coated plates for a-PM-S-MSNP and various controls. The original 96-well plate image was provided in figure 3a and 3b. Each treatment was repeated six times (n = 6). The short horizontal lines indicate mean ± SD (n = 6). For the significance analysis one-way ANOVA was performed. *$p < 0.05$, **$p < 0.01$, ***$p < 0.001$.

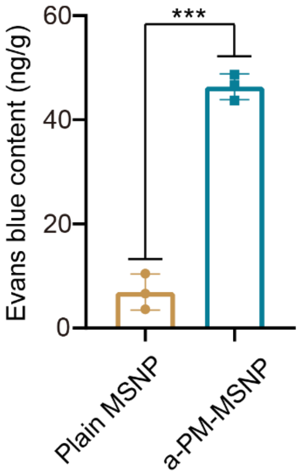

Figure S8. Quantification of Evans blue contents in the margin region from different treatment groups. The Evans blue in margin tissues was extracted with formamide for four days and the content of Evans blue was measured via UV–vis spectrophotometer at 620 nm. Each symbol represents an individual experiment (n = 3), the short horizontal lines indicate mean ± SD. For the significance analysis t-test was performed. *$p < 0.05$, **$p < 0.01$, ***$p < 0.001$.

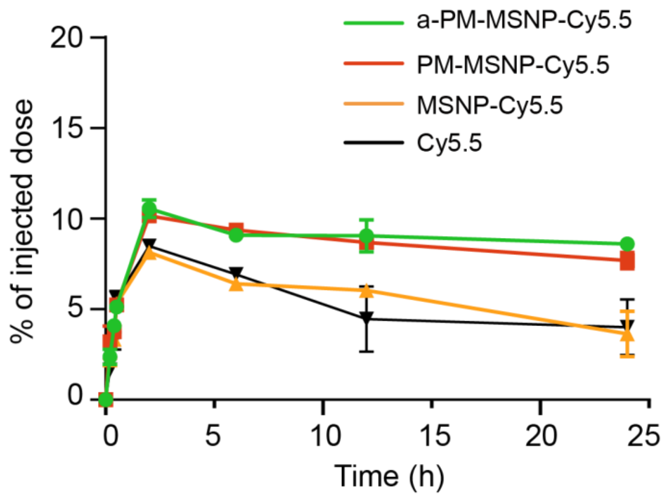

Figure S9. Pharmacokinetic curves of different formulation nanoparticles in the blood samples (quantified fluorescence intensities). Data are presented as mean ± SD (n = 3).

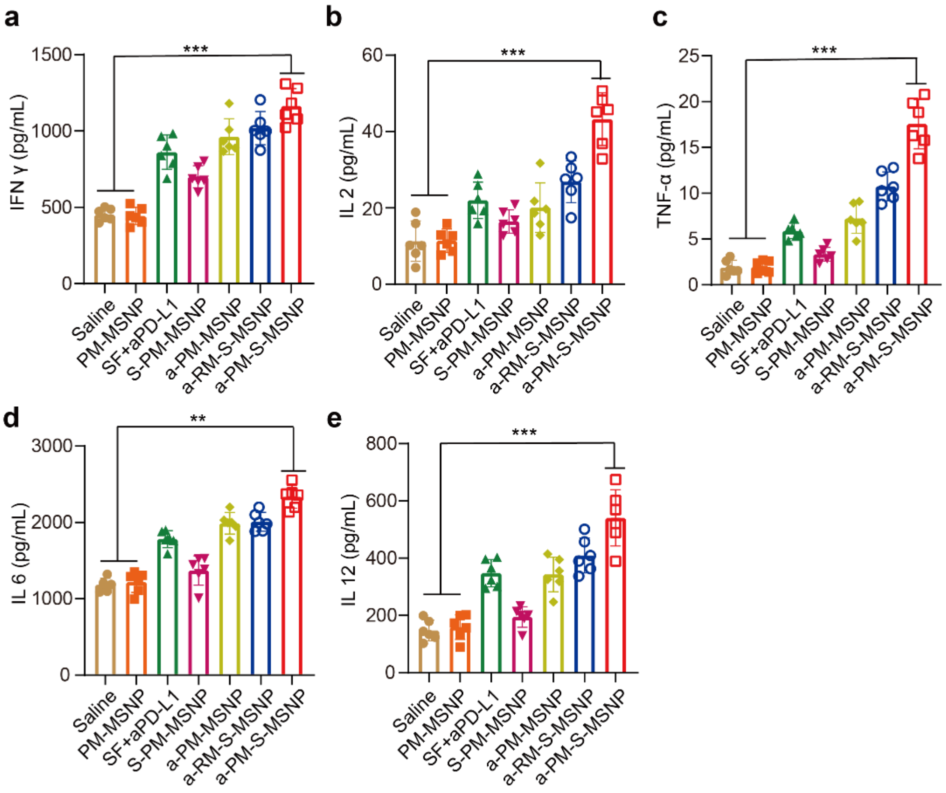

Figure S10. Pro-inflammatory cytokines and chemokines in the serum were measured using the Luminex bead-based ELISA kit (n = 6) after different formulation agent treatment. The data are presented as the means ± SD. For the significance analysis one-way ANOVA was performed. *$p < 0.05$, **$p < 0.01$, ***$p < 0.001$.

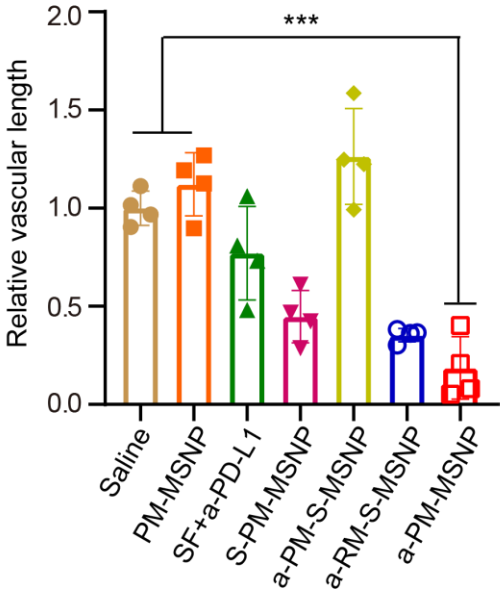

Figure S11. Quantitative analysis of the tumor vessel length of after different formulation drugs treatment (n = 3). Error bars represent the means ± SD. For the significance analysis one-way ANOVA was performed. *$p < 0.05$, **$p < 0.01$, ***$p < 0.001$.

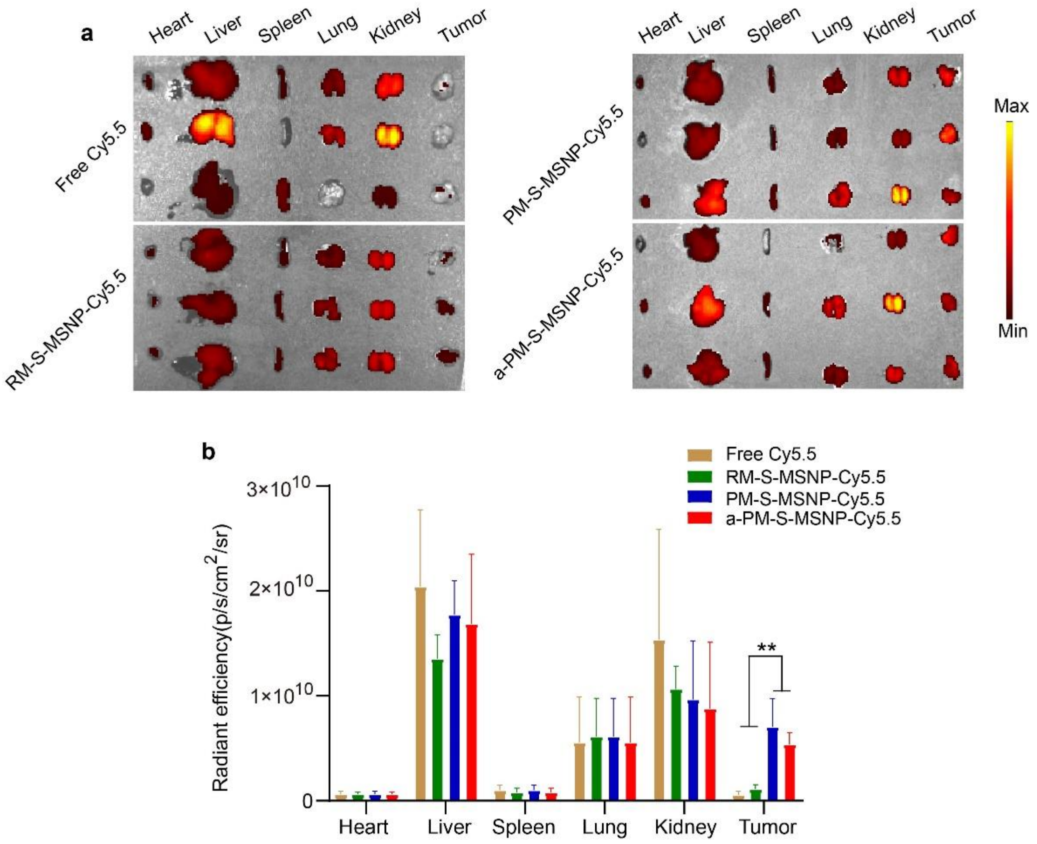

Figure S12. Pilot biodistribution study in Hep1-6 subcutaneous (subQ) tumor bearing mice. We established a surgical removal model using subQ Hep1-6 tumors, followed by different intravenous (IV) treatments. To evaluate the biodistribution profile, different Cy5.5-labelled nanoparticles were injected intravenously (IV) at Cy5.5 dose of 100 nmol/kg. a. The residual tumors and major organs were excised 12 hours after injection, and tissue fluorescence was assessed using an IVIS Spectrum Imaging System (n = 3). b. Quantitative analysis of the fluorescence intensity of organs and tumors. a-PM-S-MSNP exerted excellent residual tumor targeting ability; these particles also distributed in liver and kidney. The short horizontal lines indicate mean ± SD (n = 3). For the significance analysis one-way ANOVA was performed. $*p < 0.05$, $**p < 0.01$, $***p < 0.001$.

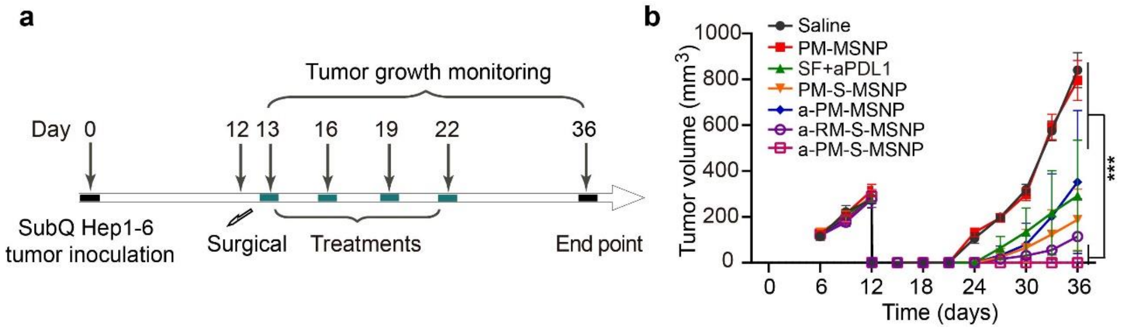

Figure S13. Antitumor efficacy of a-PM-MSNP in Hep1-6 subcutaneous tumor bearing models. a. Scheme of the animal experiment design. Briefly, tumor was implantated on day 0 and the tumor growth was monitored regularly using a caliper ruler. SubQ tumor was surgically removed on day 12 and a total of four IV injections of a-PM-S-MSNP (Sorafenib: 30 mg/kg; anti-PDL1: ~10 mg/kg) were performed every 3 days from day 13 to day 22 (n = 6). Control groups, such as free drug mixture and single drug loaded particles were included for comparison. b. Tumor growth curves up to 36 days. Tumor volume (mm^3) was calculated by the formula: (long diameter × short diameter^2)/2. Statistical significance was calculated by one-way ANOVA (n = 6). *p < 0.05, **p < 0.01, and ***p < 0.001.

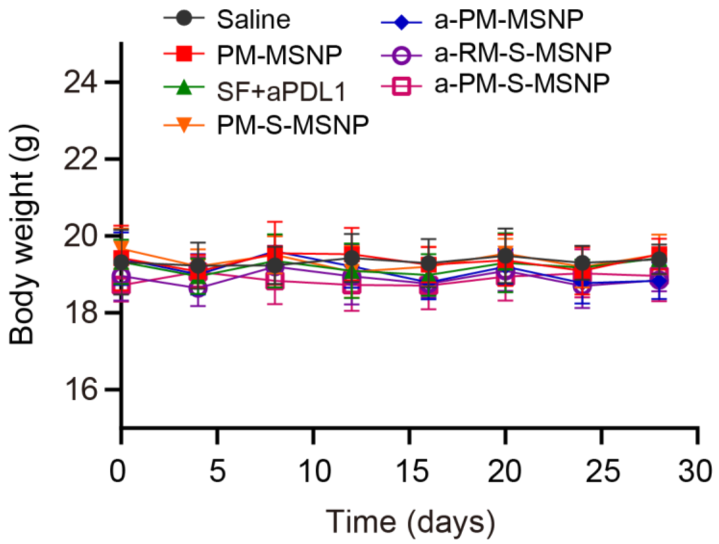

Figure S14. The body weight change of the mice during the treatment process. The short horizontal lines indicate mean ± SD (n = 6).

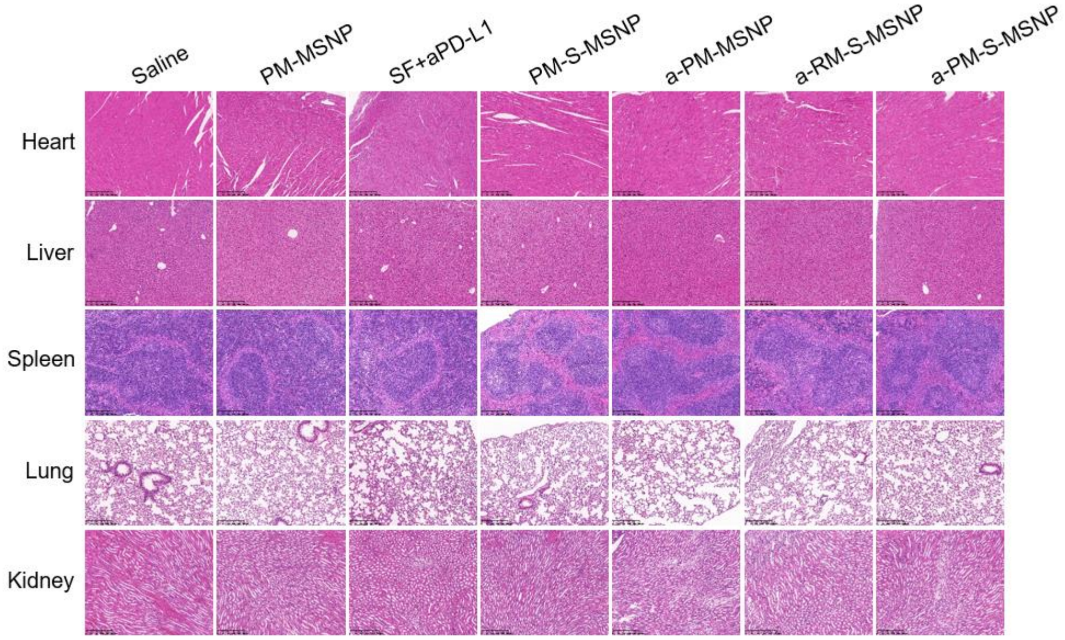

Figure S15. H&E staining of major organs of healthy mice receiving a total of three IV injections at equivalent dose of treatments described in Fig. S13 (n = 4).

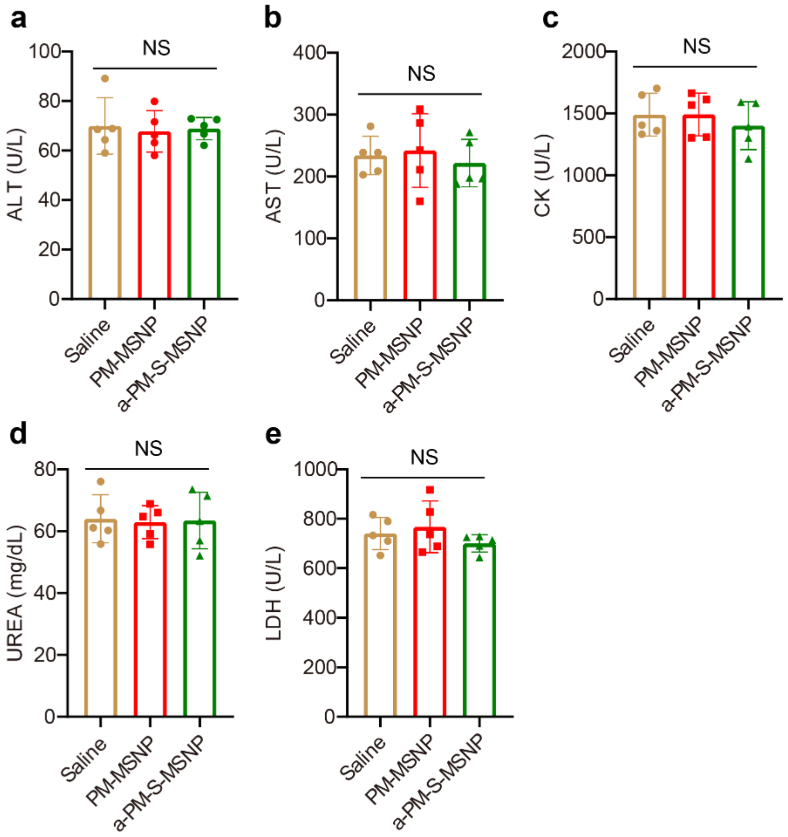

Figure S16. Serum biochemistry results of healthy mice treated with different formulation nanoparticles. ALT (a), AST (b), CK (c), UREA (d) and LDH (e) in healthy mice serum were exhibited after treatment (n = 5). Each symbol represents an individual mouse and the significance analyses were performed using one-way ANOVA. $*p < 0.05$, $**p < 0.01$, $***p < 0.001$.

Table S1. Preoperative characteristics and survival outcomes of HCC patients with different resection margin (n = 232).

Variables	High risk (n=77)	Low risk (n=155)	*P* value
**Age ≤ 60 years, N (%)**	39 (50.6)	84 (54.2)	**0.611**
**Male, N (%)**	65 (84.4)	131 (84.5)	**0.984**
**BMI, mean (SD), kg/m^2**	24.9 (21.9, 27.1)	23.9 (22.3, 26.1)	**0.248**
**ASA grade ≤ II, N (%)**	69 (89.6)	139 (89.7)	**0.987**
**Diabetes mellitus, N (%)**	16 (20.8)	32 (20.6)	**0.981**
**Smoking, N (%)**	21 (27.3)	52 (33.5)	**0.332**
**Alcohol use, N (%)**	15 (19.5)	25 (16.1)	**0.525**
**Preoperative Hb, median (IQR), g/L**	142.0 (129.0, 151.5)	142.0 (129.0, 150.0)	**0.628**
**Preoperative BG, median (IQR), mmol/L**	5.0 (4.7,5.7)	4.9 (4.5, 5.4)	**0.239**
**Preoperative ALB, median (IQR), g/L**	41.7 (39.2, 43.5)	41.4 (39.0, 44.1)	**0.772**
**Preoperative ALT, median (IQR), U/L**	36.8 (27.1, 49.6)	36.9 (24.5, 51.9)	**0.960**
**Preoperative TBIL, median (IQR), μmol/L**	12.9 (11.1, 18.1)	12.6 (9.5, 17.1)	**0.222**
**HBeAg (+), N (%)**	53 (68.8)	114 (73.5)	**0.451**
**HBV DNA ≤104 IU/mL, N (%)**	61 (79.2)	115 (74.2)	**0.399**
**Preoperative CA19-9, median (IQR), U/mL**	21.3 (10.3, 42.0)	19.7 (11.5, 36.7)	**0.678**
**Preoperative CEA, median (IQR), ng/mL**	2.3 (1.5, 3.2)	2.6 (1.8, 3.8)	**0.124**
**Preoperative AFP, median (IQR), ug/L**	131.0 (5.9, 1210.0)	89.4 (5.0, 956.9)	**0.475**
**Median RFS (95% CI), months**	17.7 (10.7-24.7)	45.8 (24.9-66.7)	**<0.001**
**Median OS (95% CI), months**	35.6 (26.7-44.5)	61.3 (49.2-73.4)	**0.001**

SD, standard deviation; IQR, interquartile range; BMI, Body Mass Index; ASA grade, American Society of Anesthesiologists physical status classification; Hb, hemoglobin; BG, blood glucose; ALB, albumin; TBIL, total bilirubin; HBV, hepatitis B virus; CA19-9, carbohydrate antigen19-9; CEA, carcinoembryonic antigen; AFP, alpha fetoprotein; RFS, recurrence-free survival; OS, overall survival; CI, confidence interval.

Table S2. Assessment of sorafenib and anti-PDL1 loading in a-PM-S-SMNP (n = 3).

Sorafenib DLR (%)	Anti-PDL1 DLR (%)
18.84	6.27
For the drug loading of sorafenib, the ratio of sorafenib to plain MSNP is 2:1 (mass ratio). For the covalently link of anti-PDL1, the ratio of anti-PDL1 to PM-S-MSNP is 10:1 (protein mass ration).	

References

[1] a) H. Meng, M. Xue, T. Xia, Z. Ji, D. Y. Tarn, J. I. Zink, A. E. Nel, *ACS Nano* **2011**, *5* (5), 4131, https://doi.org/10.1021/nn200809t; b) B. Li, T. Chu, J. Wei, Y. Zhang, F. Qi, Z. Lu, C. Gao, T. Zhang, E. Jiang, J. Xu, J. Xu, S. Li, G. Nie, *Nano Lett* **2021**, *21* (6), 2588, https://doi.org/10.1021/acs.nanolett.1c00168.

[2] a) J. Xu, Y. Zhang, J. Xu, G. Liu, C. Di, X. Zhao, X. Li, Y. Li, N. Pang, C. Yang, Y. Li, B. Li, Z. Lu, M. Wang, K. Dai, R. Yan, S. Li, G. Nie, *Adv Mater* **2020**, *32* (4), e1905145, https://doi.org/10.1002/adma.201905145; b) G. Liu, X. Zhao, Y. Zhang, J. Xu, J. Xu, Y. Li, H. Min, J. Shi, Y. Zhao, J. Wei, J. Wang, G. Nie, *Adv Mater* **2019**, *31* (32), e1900795, https://doi.org/10.1002/adma.201900795.
